# Synchronous fluorescence as a green and selective tool for simultaneous determination of bambuterol and its main degradation product, terbutaline

**DOI:** 10.1098/rsos.181359

**Published:** 2018-10-31

**Authors:** Samah Abo El Abass, Heba Elmansi

**Affiliations:** 1 Department of Pharmaceutical Analytical Chemistry, Faculty of Pharmacy, Mansoura University, 35516 Mansoura, Egypt; 2 Department of Pharmaceutical Chemistry, Faculty of Pharmacy, Delta University for Science and Technology, Gamasa 35715, Egypt

**Keywords:** terbutaline, bambuterol, derivative, synchronous, spectrofluorimetric

## Abstract

A green, sensitive and cost-effective method is introduced in this research for the determination of bambuterol and its main degradation product, terbutaline, simultaneously, relying on the synchronous spectrofluorimetric technique. First derivative synchronous spectrofluorimetric amplitude is measured at Δ*λ* = 20 nm, so bambuterol can be quantitated at 260 nm, and terbutaline can be measured at 290 nm, each at the zero crossing point of the other. The amplitude–concentration plots were linear over the concentration ranges of 0.2–6.0 µg ml^−1^ and 0.2–4.0 µg ml^−1^ for both bambuterol and terbutaline, respectively. Official guidelines were followed to calculate the validation parameters of the proposed method. The low values of limits of detection of 0.023, 0.056 µg ml^−1^ and limits of quantitation of 0.071, 0.169 µg ml^−1^ for bambuterol and terbutaline, respectively, point to the sensitivity of the method. Bambuterol is a prodrug for terbutaline, and the latter is considered its degradation product so the established method could be regarded as a stability-indicating one. Moreover, the proposed method was used for the analysis of bambuterol and terbutaline in their single ingredient preparations and the results revealed statistical agreement with the reference method. The suggested method, being a simple and low-cost procedure, is superior to the previously published methods which need more sophisticated techniques, longer analysis time and highly toxic solvents and reagents. It could be considered as an eco-friendly analytical procedure.

## 1. Introduction

One of the most common types of photoluminescence in analytical chemistry is fluorescence spectroscopy and it is also the most useful because of its sensitivity and simplicity. The type of fluorimetry that allows simultaneous scanning for the excitation and the emission wavelengths with a small wavelength difference between them is known as synchronous fluorimetry [1]. Synchronous fluorimetry is useful in the analysis of mixtures [2] which in most cases have overlapped excitation and/or emission spectra, due to its advantages which include improved selectivity over normal excitation emission fluorimetry, diminishing the interference of scattering light, providing faster measurement and more simple spectra [3]. Combining synchronous fluorescence spectroscopy and derivative amplitude results in more advantages, as the derivative signal amplitude is inversely proportional to the band width of the original spectrum [3]. Based on this, the first derivative technique offers the ability for multicomponent resolution, and for determination of compounds in the presence of impurities or interferences [4].

**Figure 1.**
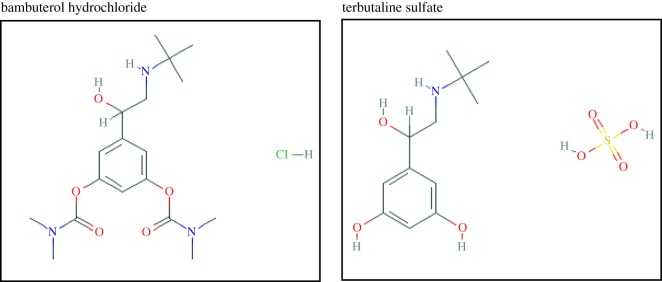
Chemical structures of bambuterol hydrochloride and terbutaline sulfate.

The beta2-adrenoceptor agonist bambuterol hydrochloride (BAM) is an official drug in the British Pharmacopoeia (BP) [5] (figure 1), and is chemically named as 5-[(1*RS*)-2-[(1,1-dimethylethyl)amino]-1-hydroxyethyl]-1,3-phenylenebis(dimethylcarbamate) hydrochloride. Terbutaline sulfate (TEB) is also a beta2-adrenoceptor agonist [5] and is named as bis[(1*RS*)-1-(3,5-dihydroxyphenyl)-2-[(1,1-dimethylethyl)amino]ethanol] sulfate [5] (figure 1). Bambuterol is an inactive prodrug of terbutaline, a direct sympathomimetic with mainly beta-adrenergic activity and a selective action on beta2 receptors. It has similar actions to those of salbutamol except that it has a more prolonged duration of action (at least 24 h) [6]. The ideal stability-indicating method is that one has the ability to determine the compound of importance alone and in the presence of its degradation product [7]. Research papers which deal with simultaneous determination of BAM and TEB are based mainly on mass spectrometry and gas chromatography [8–10]. There is one research depending on spectrophotometric ratio spectra in quantitation of BAM in the presence of TEB [11], but neither conventional nor synchronous spectrofluorimetry has been used before for their binary mixture analysis.

For the previously mentioned advantages of the derivative synchronous technique, we aimed to determine BAM and TEB simultaneously, for the first time, using these merits in their analysis and avoiding other high-cost techniques. The proposed method is a simple method without pretreatment steps, and in addition to its sensitivity, it does not need any sophisticated techniques and depends on a direct measurement step which makes it more advantageous. It would be useful especially when low-cost analysis is preferred.

## 2. Experimental set-up

### 2.1. Apparatus

Cary Eclipse fluorescence spectrophotometer from Agilent Technologies and Cary Eclipse software were used for measuring synchronous fluorescence spectra at Δ*λ* = 20, smoothing factor = 15 and slit width = 5 nm during all the experiments. The instrument was adjusted at high sensitivity mode (800 v).

For the reference method, the high-performance liquid chromatography (HPLC) system used was a Merck Hitachi L-7100 chromatograph equipped with a Rheodyne injector valve with a 20 µl loop and a L-7400 UV detector (Darmstadt, Germany).

For measuring and adjusting the pH, a Consort NV P-901 pH meter (Belgium) was used.

### 2.2. Materials and reagents

— BAM was kindly provided by AstraZeneca Pharmaceutical Egypt. The purity was found to be 99.98% as informed by the manufacturer.
— TEB was kindly provided by SEDICO, Egypt, with purity 99.97% as labelled by the company.
— Pharmaceutical preparations:
  1. Bambec^®^ tablet: labelled to contain BAM (10 mg tablet^−1^), manufactured by AstraZeneca Egypt under licence of AstraZeneca Sweden.
  2. Bambedil^®^ syrup: contains BAM (5 mg/5 ml), the product of Western Pharmaceutical Industries.
  3. Aironyl^®^ tablet: labelled to contain TEB (2.5 mg tablet^−1^), product of SEDICO, 6 October City, Egypt.
  4. Aironyl^®^ syrup: labelled to contain TEB (1.5 mg/5 ml), product of SEDICO, 6 October City, Egypt.
— Acetonitrile, methanol, ethanol and propanol were purchased from Sigma Aldrich (Germany).
— Acetic acid, sodium acetate, sodium hydroxide, hydrochloric acid, borax and boric acid were purchased from El-Nasr Pharmaceutical Chemicals Company, Cairo, Egypt.
— Sodium octanesulfonate, for the reference method, was purchased from Sigma Aldrich (Germany).

### 2.3. Preparation of stock solutions

Stock solution equivalent to 100.0 µg ml^−1^ of BAM or 100.0 µg ml^−1^ of TEB was prepared separately by dissolving 10.0 mg of either BAM or TEB in a 100 ml volumetric flask and completed to volume with water. The stock solution was further diluted by taking 10.0 ml from it into the 100 ml volumetric flask and completing the volume with water to get a diluted stock solution with concentration 10 µg ml^−1^.

## 3. General procedures

### 3.1. Calibration curve construction

Aliquots of appropriate standard solutions of BAM and TEB covering the concentration ranges of 0.2–6.0 and 0.2–4.0 µg ml^−1^, respectively, were taken into a 10 ml volumetric flasks set and diluted with water. The synchronous fluorescence spectra of the samples at a constant wavelength difference Δ*λ* = 20 nm were recorded. The first derivative synchronous fluorescence spectra of the two drugs were then derived using Cary Eclipse software and the peak amplitudes of the first derivative spectra (^1^D) were measured at 260 nm and 290 nm for BAM, and TEB, respectively, in parallel with the blank experiment. Regression equations were derived by plotting the peak amplitude of the first derivative spectra (^1^D) against the final concentration of each drug in µg ml^−1^ to get the calibration graphs.

### 3.2. Synthetic mixtures and pharmaceutical preparation analysis

Synthetic mixtures were prepared by taking aliquots from each drug to reach final concentrations of (2.00 + 0.20 µg ml^−1^), (4.00 + 0.40 µg ml^−1^), (2.00 + 1.00 µg ml^−1^), (2.00 + 2.00 µg ml^−1^) and (5.00 + 1.00 µg ml^−1^) of BAM and TEB, respectively, the solutions were completed to the volume with water and mixed well. The procedure for calibration curve construction was then carried out. The peak amplitudes of the first derivative synchronous spectra (^1^D) corresponding to each drug in their different ratios were measured simultaneously. From these data, the percentage recoveries of the drug were then calculated.

For pharmaceutical preparations, ten Bambec^®^ or Aironyl^®^ tablets were weighed, and finely pulverized. Accurately weighed quantity corresponding to 10.0 mg BAM or TEB was transferred separately into 100 ml volumetric flasks and approximately 70 ml water was added. The contents of each flask were sonicated for 30 min, completed to the mark with water. The solutions were filtered into 100 ml volumetric flasks. Further dilution was carried out with water. In the case of syrups, a volume of Bambedil^®^ or Aironyl^®^ syrup equivalent to 10.0 mg BAM or TEB, respectively, was diluted to 100 ml with distilled water in a volumetric flask. The solutions were subjected to further dilution with water to obtain a working standard solution. The procedure for calibration curve construction was then followed and the content of tablets was calculated through the correlative regression equation.

## 4. Results and discussion

**Figure 2.**
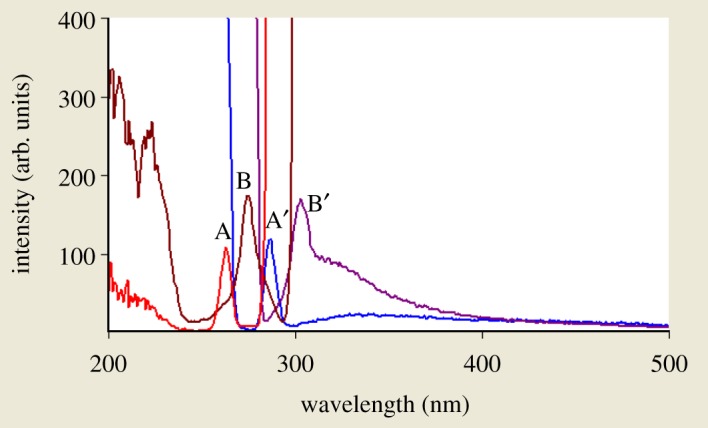
Excitation and emission spectra of BAM and TEB in water where: A, A′ are BAM excitation and emission spectra at concentration (0.5 µg ml^−1^) and B, B′ are TEB excitation and emission spectra at concentration (0.5 µg ml^−1^).

**Figure 3.**
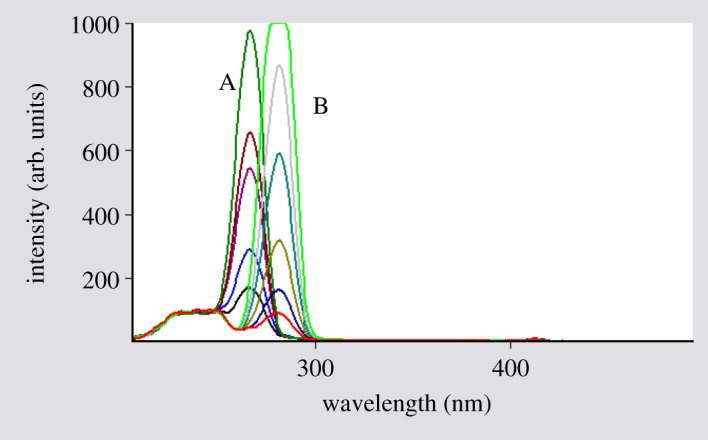
Synchronous spectra for BAM (A) and TEB (B), which indicate overlapping spectra.

**Figure 4.**
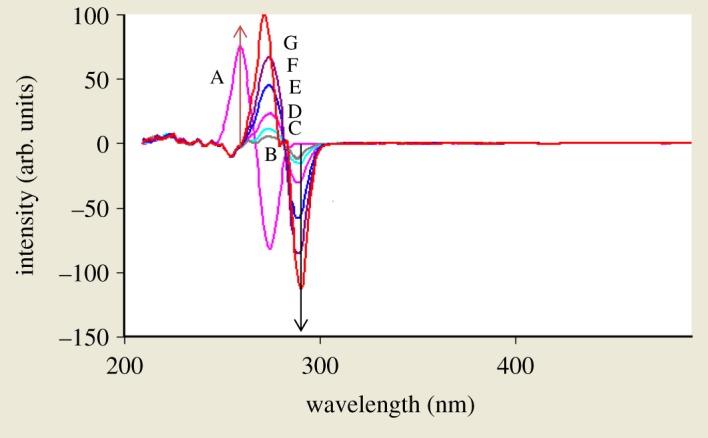
First derivative amplitudes of synchronous fluorescence spectra of BAM and different concentrations of TEB at 260 nm and 290 nm, respectively, where A is BAM at concentration of 4 µg ml^−1^. B–G are TEB at concentrations of (0.2, 0.4, 0.8, 1.6, 3.2 and 4.0 µg ml^−1^).

**Figure 5.**
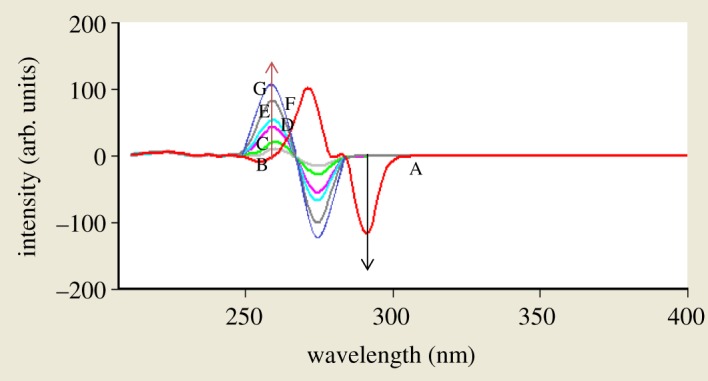
First derivative amplitudes of synchronous fluorescence spectra of different concentrations of BAM and TEB at 260 nm and 290 nm, respectively, where A is TEB at concentration of 4 µg ml^−1^. B–G are BAM at concentrations of (0.2, 0.8, 1.6, 3.2, 4.0 and 6.0 µg ml^−1^).

When dealing with a mixture of fluorescent components, the beneficial effect of synchronous scanning is to greatly simplify the spectrum and decrease the extent of spectral overlaps [12] as synchronous scanning is often helpful in resolving overlapping absorption and fluorescence spectra of mixtures of pharmaceutical compounds. The excitation–emission fluorescence spectra of BAM and TEB show overlapping in a manner that prevents simultaneous determination of them without interference (figure 2). This led us to apply synchronous scanning to solve such problem, but even synchronous spectra did not permit measuring the two drugs simultaneously as shown in figure 3; although there was a point that TEB could be measured in the presence of BAM at 290 nm, but with increasing concentrations this point was distorted by slight interference from BAM spectrum. BAM could not be measured due to the presence of interference from TEB at 260 nm which did not permit its accurate measurement. By measuring first derivative amplitudes of synchronous fluorescence spectra of BAM and TEB at 260 and 290 nm, respectively, we can simply solve the problem of overlapping spectra and measure each drug in the presence of the other (figures 4 and 5) without addition of any reagents and without prior treatment steps using the zero crossing point concept in which the value of the first derivative for one drug is zero, although the concentration of the other drug varies. Consequently, the first derivative amplitude is dependent only on the concentration of the compound [13]. This concept has exhibited great efficacy for multicomponent analysis [13], and for assay of compounds in the presence of impurities, degradation products or other drugs that may interfere [4,14].

### 4.1. Optimization of different parameters

Different variables which would affect the sensitivity and precision of the method were studied and optimized.

**Figure 6.**
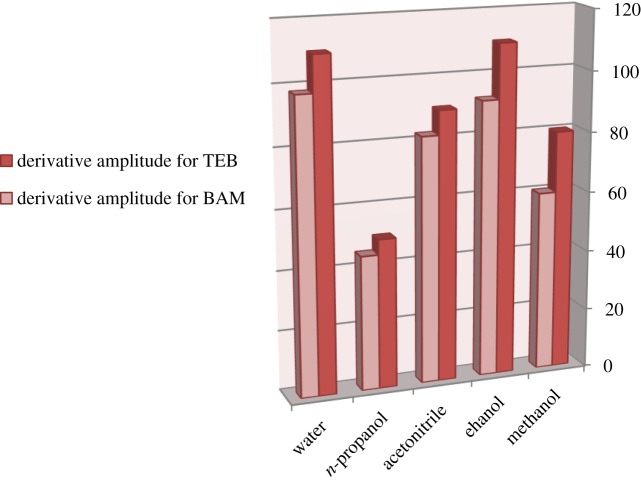
Effect of diluting solvents on the first derivative amplitudes of synchronous fluorescence spectra of BAM and TEB (4 µg ml^−1^ each).

The effect of diluting solvents was studied using methanol, ethanol, acetonitrile and propanol in addition to water as shown in figure 6. It was found that using methanol resulted in a wavelength shift and absence of the zero crossing point for TEB. Other solvents did not make this shift, but water and ethanol gave the highest sensitivity when used. Hence, water was chosen as the optimum diluting solvent. Using water adds to the advantages of the procedure as regards greenness and simplicity.

**Figure 7.**
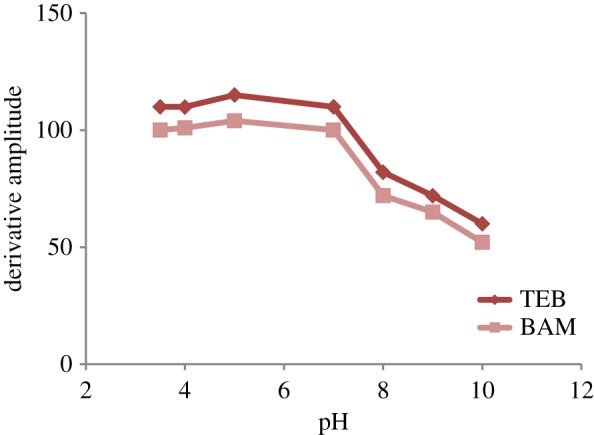
Effect of pH on the first derivative amplitudes of synchronous fluorescence spectra of BAM and TEB (4 µg ml^−1^ each).

The pH effect was also studied using 0.2 M acetate and 0.2 M borate buffers over the range (3–10). Increasing pH above 7 resulted in decreased sensitivity and deformation of the spectra shapes (shift in the wavelength which affects the zero crossing points). Acidic pH from 3 to 5 gave results comparable to using water only (figure 7). For simplicity of the proposed method, the drugs were measured in water only without the addition of any buffer.

**Figure 8.**
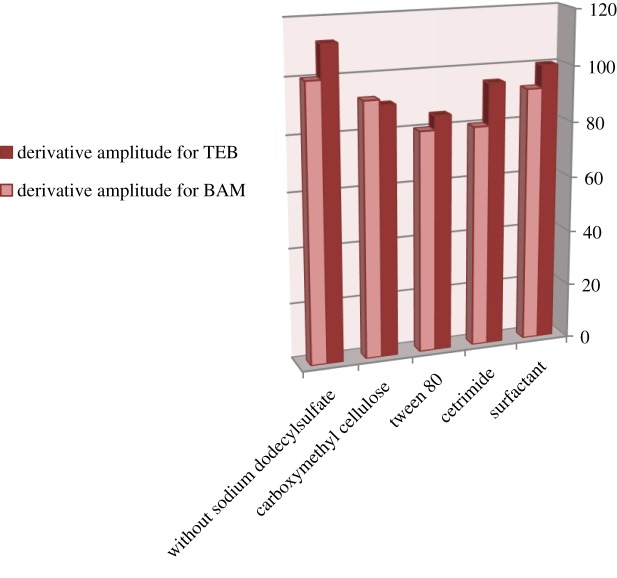
Effect of different surfactants on the first derivative amplitudes of synchronous fluorescence spectra of BAM and TEB (4 µg ml^−1^ each).

There was a trial to add micellar media seeking to increase the sensitivity of the method [15]. Different micellar systems, such as sodium dodecylsulfate (anionic surfactant), cetrimide (cationic surfactant), carboxymethyl cellulose and tween 80 (non-ionic surfactants), were added in a volume of 1 ml (0.5% w/v) for each one separately. It was found that none of the above surfactants make a distinguishable increase in the sensitivity of the method (figure 8); therefore, the study was carried out without adding any of them.

The optimum Δ*λ* is essential for obtaining good well-resolved spectra with highest possible sensitivity. Different Δ*λ* values were studied ranging from 10 nm to 100 nm. It was found that Δ*λ* = 20 nm was the optimum for this study giving the highest intensity for the two drugs with the optimum spectral shape and signal value.

### 4.2. Validation of the proposed method

Validation was evaluated regarding ICH recommendations [7] in terms of linearity, accuracy, precision and selectivity. The proposed method was found to give a linear response between the concentration and the first derivative amplitude (^1^D) of BAM and TEB over concentration ranges (0.2–6.0 µg ml^−1^) and (0.2–4.0 µg ml^−1^), respectively. The linear regression equations are: ^1^D = 18.21 + 13.65 C for BAM at 260 nm and ^1^D = 8.29 + 27.57 C for TEB at 290 nm. The calculated limits of detection and of quantitation according to ICH guidelines [7] were found to be 0.023 µg ml^−1^, 0.056 µg ml^−1^ and 0.071 µg ml^−1^, 0.169 µg ml^−1^, respectively (table 1).

**Table 1.** Analytical performance data of the proposed first derivative synchronous spectrofluorimetric method.

parameter	BAM	TEB
wavelength (nm)	260	290
number of samples (*n*)	6	6
linearity range (µg ml^−1^)	0.2–6.0	0.2–4.0
intercept (*a*)	18.21	8.29
slope (*b*)	13.65	27.57
correlation coefficient (*r*)	0.9999	0.9999
s.d. of residuals (*S_y/x_*)	0.143	0.733
s.d. of intercept (*S_a_*)	0.096	0.464
s.d. of slope (*S_b_*)	0.029	0.201
percentage relative standard deviation, % RSD	1.11	1.70
percentage relative error, % error	0.45	0.69
limit of detection, LOD (µg ml^−1^)	0.023	0.056
limit of quantitation, LOQ (µg ml^−1^)	0.0701	0.169

Table 2 shows that the proposed first derivative synchronous spectrofluorimetric method results were compared statistically with results of the official BP method [5] in three different levels of concentration, and it was found that there was no significant difference between them which was proved by Student's *t*-test and variance ratio *F*-test [16]. The reference method used was adopted by the BP [5] which suggested an HPLC method for analysing BAM in the presence of its related substances. The mobile phase consisted of 1.3 g of sodium octanesulfonate in 430 ml of a mixture of 25 volumes of acetonitrile and 75 volumes of methanol; then this solution was mixed with 570 ml of 0.050 M phosphate buffer (pH 3.0).

**Table 2.** Application of the proposed method to the determination of BAM and TEB in their pure form.

	proposed method			official method [5]	
compound	amount taken (μg ml^−1^)	amount found (μg ml^−1^)	% recovery	amount taken (μg ml^−1^)	% recovery
BAM	0.20	0.204	102.00	40.0	99.34
	0.80	0.790	98.75	60.0	100.89
	1.60	1.596	99.75	80.0	99.67
	3.20	3.207	100.22		
	4.00	4.013	100.33		
	6.00	5.989	99.83		
mean	100.15			99.97	
no. of samples	6			3	
±s.d.	1.11			0.82	
*t*	0.25 (2.36)				
*F*	1.70 (19.29)				
TEB	0.20	0.207	103.50	40.0	100.56
	0.40	0.406	101.50	60.0	99.26
	0.80	0.805	100.63	80.0	100.28
	1.60	1.585	99.06		
	3.20	3.163	98.84		
	4.00	4.034	100.85		
mean	100.73			100.03	
no. of samples	6			3	
±s.d.	1.71			0.68	
*t*	0.66 (2.36)				
*F*	6.26 (19.29)				

Each result is the average of three separate determinations. The values between parentheses are the tabulated *t* and *F* values at *p* = 0.05 [16].

The precision (repeatability and intermediate precision) of the proposed method was tested by performing inter-day and intra-day analysis on three different concentrations throughout the ranges for both BAM and TEB. High precision of the developed method was clear from the calculated values of s.d. and % RSD for both drugs which were relatively small (table 3). It is also worth mentioning that the robustness was high as there is no reagent or buffer added for which any alteration of their volumes may result in variation in the response, so the simplicity of the method added to its robustness.

**Table 3.** Inter-day and intra-day precision analysis.

		intra-day			inter-day		
drug	conc. (μg ml^−1^)	mean *±* s.d	%RSD	% error	mean *±* s.d	%RSD	% error
BAM	0.40	102.00 ± 1.33	1.34	0.67	99.25 ± 0.65	0.66	0.38
	3.20	100.17 ± 1.66	1.66	0.96	100.09 ± 0.79	0.79	0.46
	6.00	99.94 ± 1.30	1.30	0.75	101.37 ± 1.97	1.94	1.12
TEB	0.20	100.42 ± 0.81	0.81	0.46	99.42 ± 1.68	1.69	0.98
	3.20	99.86 ± 0.30	0.30	0.17	99.09 ± 1.72	1.74	1.00
	4.00	100.61 ± 1.23	1.23	0.71	99.33 ± 1.68	1.69	0.97

Each result is the average of three separate determinations.

**Figure 9.**
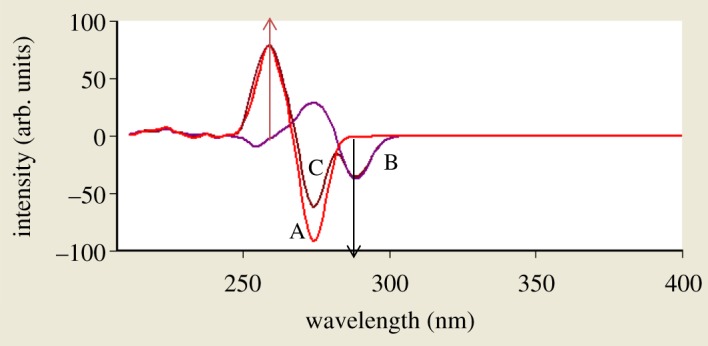
First derivative synchronous fluorescence spectra of: (A) 5 µg ml^−1^ BAM; (B) 1 µg ml^−1^ TEB; (C) synthetic mixture of both.

**Figure 10.**
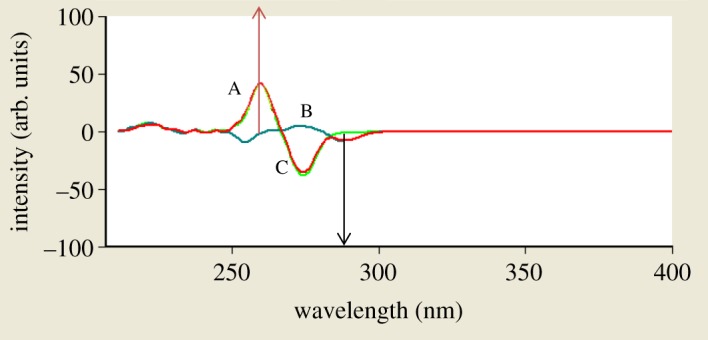
First derivative synchronous fluorescence spectra of: (A) 2 µg ml^−1^ BAM; (B) 0.2 µg ml^−1^ TEB; (C) synthetic mixture of both.

The proposed first derivative synchronous spectrofluorimetric method permits simultaneous BAM and TEB determination with no interference, ensuring its selectivity and ability to resolve the mixture of two drugs in different ratios (figures 9 and 10). Synthetic mixtures of BAM and TEB were investigated in different ratios (1 : 1, 2 : 1, 5 : 1 and 10 : 1 for BAM and TEB, respectively) and the concentrations of each drug were calculated with good percentage recoveries (table 4).

**Table 4.** Application of the proposed method for the determination of BAM and TEB in their synthetic mixtures.

	proposed method				official method [5]			
	amount taken (μg ml^−1^)		% recovery		amount taken (μg ml^−1^)		% recovery	
	BAM	TEB	BAM	TEB	BAM	TEB	BAM	TEB
	2.00	0.20	100.24	102.14	100.00	50.00	99.35	98.35
	4.00	0.40	99.62	101.32	100.00	20.00	98.62	101.24
	2.00	1.00	99.64	98.62	50.00	50.00	99.44	99.64
	5.00	1.00	101.32	99.11				
	2.00	2.00	98.37	99.32				
mean			99.84	100.10			99.14	99.74
no. of samples	5				3			
±s.d.			1.07	1.54			0.45	1.45
*t*			1.05 (2.45)	0.326 (2.45)				
*F*			5.69 (19.25)	1.125 (19.25)				

Each result is the average of three separate determinations. The values between parentheses are the tabulated *t* and *F* values at *p* = 0.05 [16].

### 4.3. Assay of bambuterol and terbutaline in their pharmaceutical preparations

The proposed method was applied to the determination of the commercial tablets and syrups of both BAM and TEB and the results were satisfactory. Specificity is also proved through the analysis of the pharmaceutical preparations, as it was found that the common tablet and syrup excipients did not interfere significantly with the results of the proposed method as indicated by good percentage recoveries obtained (table 5). The excipients which are present in tablets for the studied pharmaceutical preparations included: lactose monohydrate, maize starch, povidone, microcrystalline cellulose, magnesium stearate and purified water. The syrup excipients including sucrose in water were combined with the flavouring agent.

**Table 5.** Application of the proposed method for the determination of BAM and TEB in their pharmaceutical preparations.

	proposed method			official method [5]		
compound	amount taken (μg ml^−1^)	amount found (μg ml^−1^)	% recovery	amount taken (μg ml^−1^)	amount found (μg ml^−1^)	% recovery
Bambec^®^ tablet	1.00	1.026	102.60	20.00	20.376	101.88
	2.50	2.447	97.88	40.00	39.185	97.96
	4.00	4.027	100.68	60.00	60.345	100.58
mean			100.39			100.14
no. of samples	3			3		
±s.d.			2.37			2.00
*t*	0.137 (2.77)					
*F*	1.413 (19.00)					
Aironyl^®^ tablet	1.25	1.272	101.76	12.50	12.655	101.24
	2.50	2.456	98.24	25.00	24.692	98.77
	4.00	4.024	100.59	40.00	40.164	100.41
mean			100.20			100.14
no. of samples	3			3		
±s.d.			1.79			1.26
*t*	0.045 (2.77)					
*F*	2.034 (19.00)					
Bambedil^®^ syrup	1.00	0.983	98.30	20.00	20.44	102.2
	2.50	2.535	101.40	40.00	39.98	99.95
	4.00	3.983	99.58	60.00	19.84	99.22
mean			99.76			100.46
no. of samples	3			3		
±s.d.			1.56			1.55
*t*	0.55 (2.77)					
*F*	1.00 (19.00)					
Aironyl^®^ syrup	1.25	1.262	100.96	12.50	12.49	99.98
	2.50	2.476	99.04	25.00	25.39	101.54
	4.00	4.013	100.32	40.00	39.47	98.67
mean			100.11			100.06
no. of samples	3			3		
±s.d.			0.98			1.44
*t*	0.043 (2.77)					
*F*	2.16 (19.00)					

The values between parentheses are the tabulated *t* and *F* values at *p* = 0.05 [16].

## 5. Conclusion

Establishing an accurate and precise green analytical method for multicomponent analysis using time-and cost-effective procedures is greatly advantageous. For the first time, a sensitive and simple first derivative synchronous spectrofluorimetric method has been investigated for the simultaneous determination of BAM and TEB synthetic mixtures. Considering its simplicity and sensitivity, the proposed method permits the analysis of BAM and TEB in different ratios up to 10 : 1, respectively, so it could be applied in quality control laboratories. The method permits assaying BAM and TEB in different tablet and syrup formulations without excipient interference and the results obtained were favourably compared to those obtained with the reference pharmacopoeial HPLC method. In terms of green analysis, the method is regarded a good alternative to the reported methods using hazardous non-degradable chemicals and large amounts of organic solvents.

## Supplementary Material

Graphical Abstract

## Data accessibility

Data are available at Dryad Digital Repository: http://dx.doi.org/10.5061/dryad.784vv04 [17].
