# Supplementary material for: Synchronous fluorescence as a green and selective tool for simultaneous determination of bambuterol and its main degradation product, terbutaline
Source: R Soc Open Sci. 2018 Oct 31;5(10):181359. doi: 10.1098/rsos.181359 (PMC6227927; doi:10.1098/rsos.181359)
Supplement: Graphical Abstract [file rsos181359supp1.pdf]

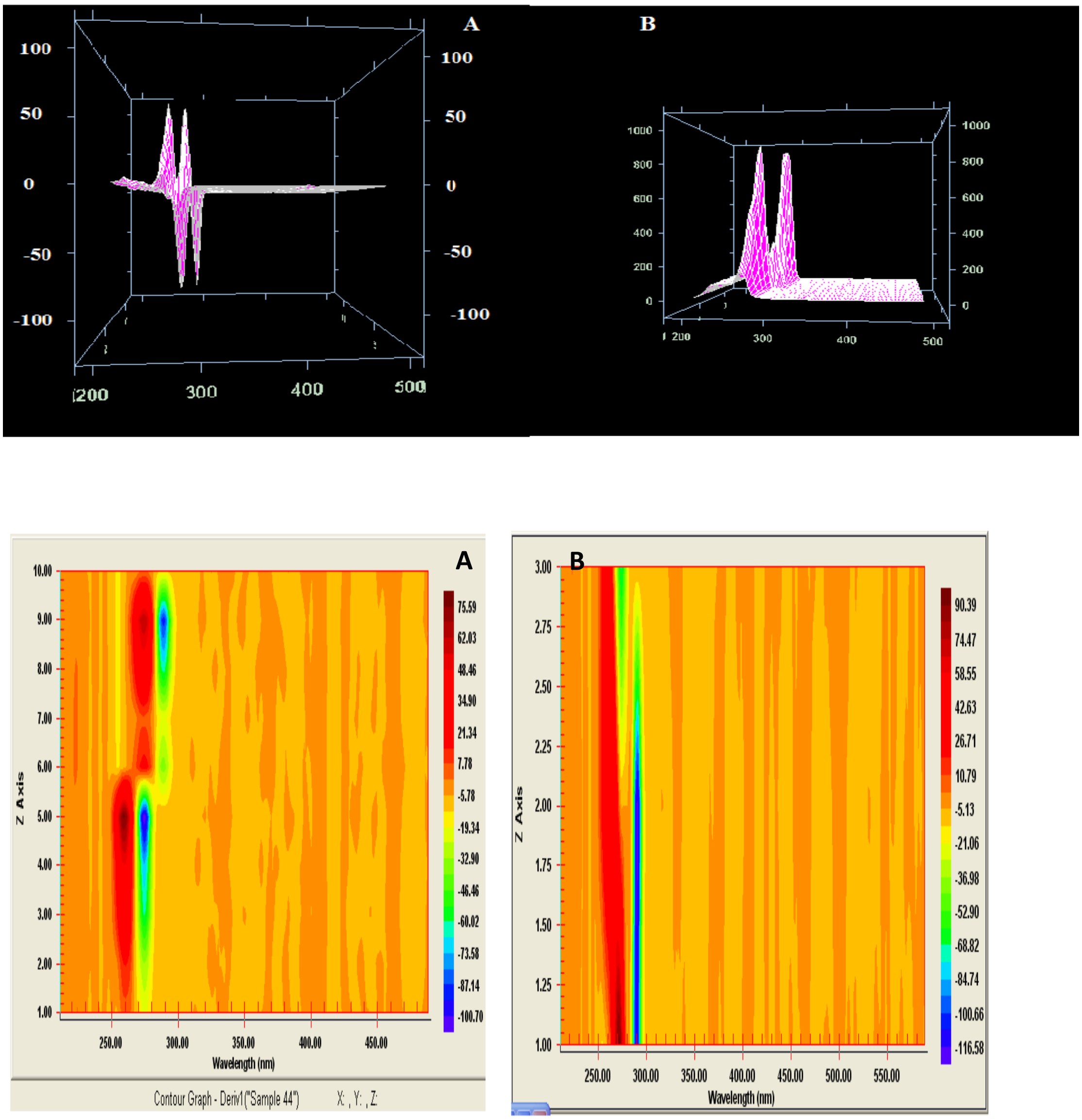

A is 3 D and contour plot of first derivative spectra of BAM and TEB

B is3 D and contour plot of synchronous spectra of BAM and TEB
